# Infectious Flexor Tenosynovitis of the Lower Extremity: A Case Report

**DOI:** 10.5070/M5.52282

**Published:** 2025-10-31

**Authors:** Daniela Usuga, Dustin Harris

**Affiliations:** * UT Southwestern, Department of Emergency Medicine, Dallas, TX

## Abstract

Infectious flexor tenosynovitis (IFT) of the lower extremity is rare but serious, requiring early recognition and aggressive management to prevent severe morbidity. Often associated with penetrating trauma or hematogenous spread, IFT can lead to devastating complications if not promptly identified and treated.

A 57-year-old female with cirrhosis, diabetes mellitus, and hypertension presented to the emergency department with progressive right ankle pain, swelling, and systemic symptoms. Despite prior emergency visits, her condition worsened. Imaging revealed infectious tenosynovitis involving the flexor hallucis longus and flexor digitorum longus tendons, with concerning signs of soft tissue necrosis. She received broad-spectrum antibiotics and underwent emergent surgery, ultimately requiring a below-the-knee amputation due to extensive necrosis. Blood cultures confirmed *group B Streptococcus* as the pathogen. Her postoperative course was complicated by hepatic encephalopathy and hematochezia, necessitating multidisciplinary care.

This case highlights the importance of early recognition of IFT, particularly in immunocompromised patients, and the role of imaging in diagnosing deep soft tissue infections. Delayed diagnosis can lead to severe outcomes, emphasizing the need for heightened clinical suspicion and timely intervention. Increased awareness and prompt management of IFT are crucial for improving patient outcomes and limb preservation.

**Topics:**

Infectious flexor tenosynovitis, flexor hallucis longus, flexor digitorum longus, Emergency Department.

**Figure:**
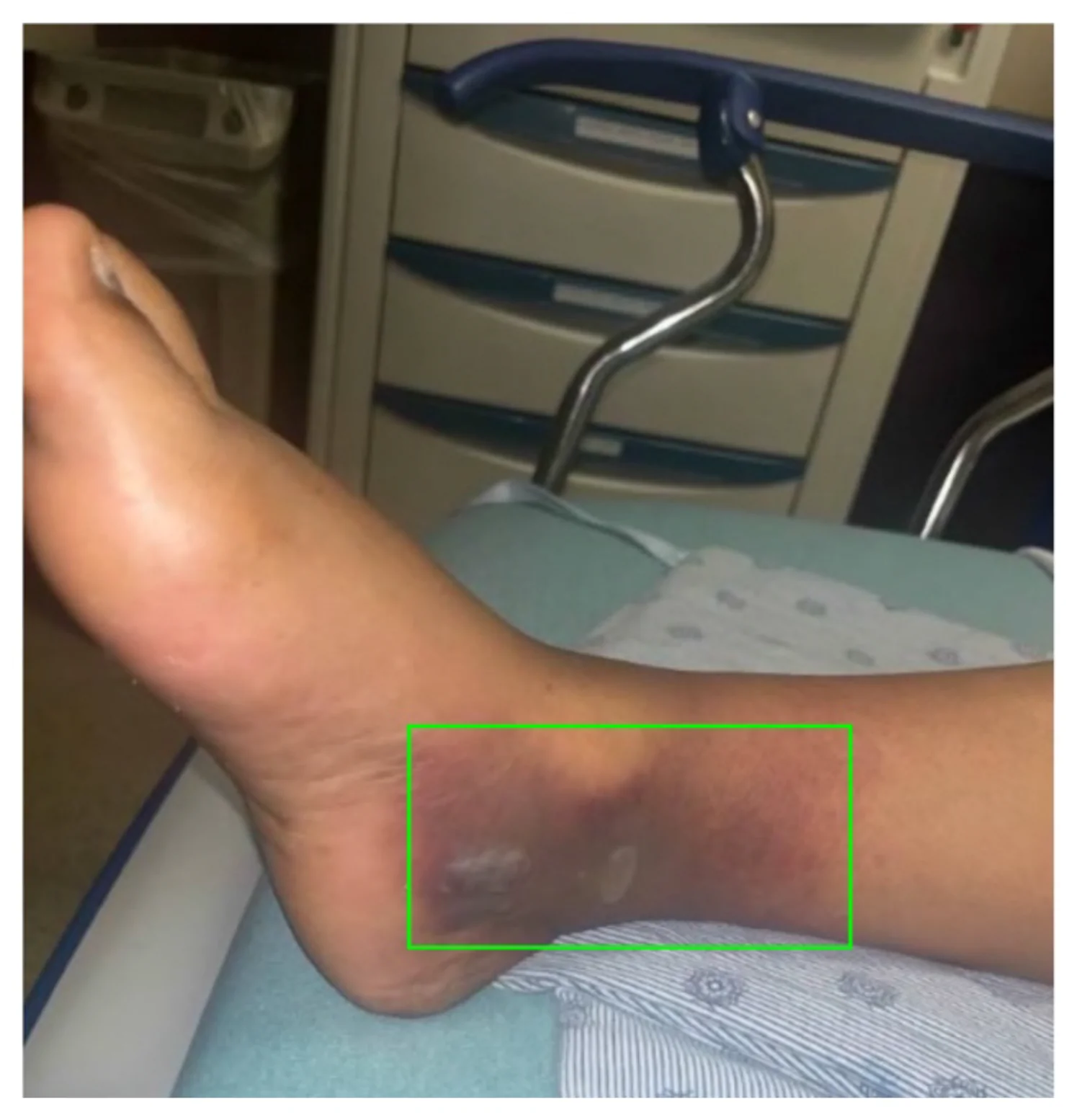


**Figure:**
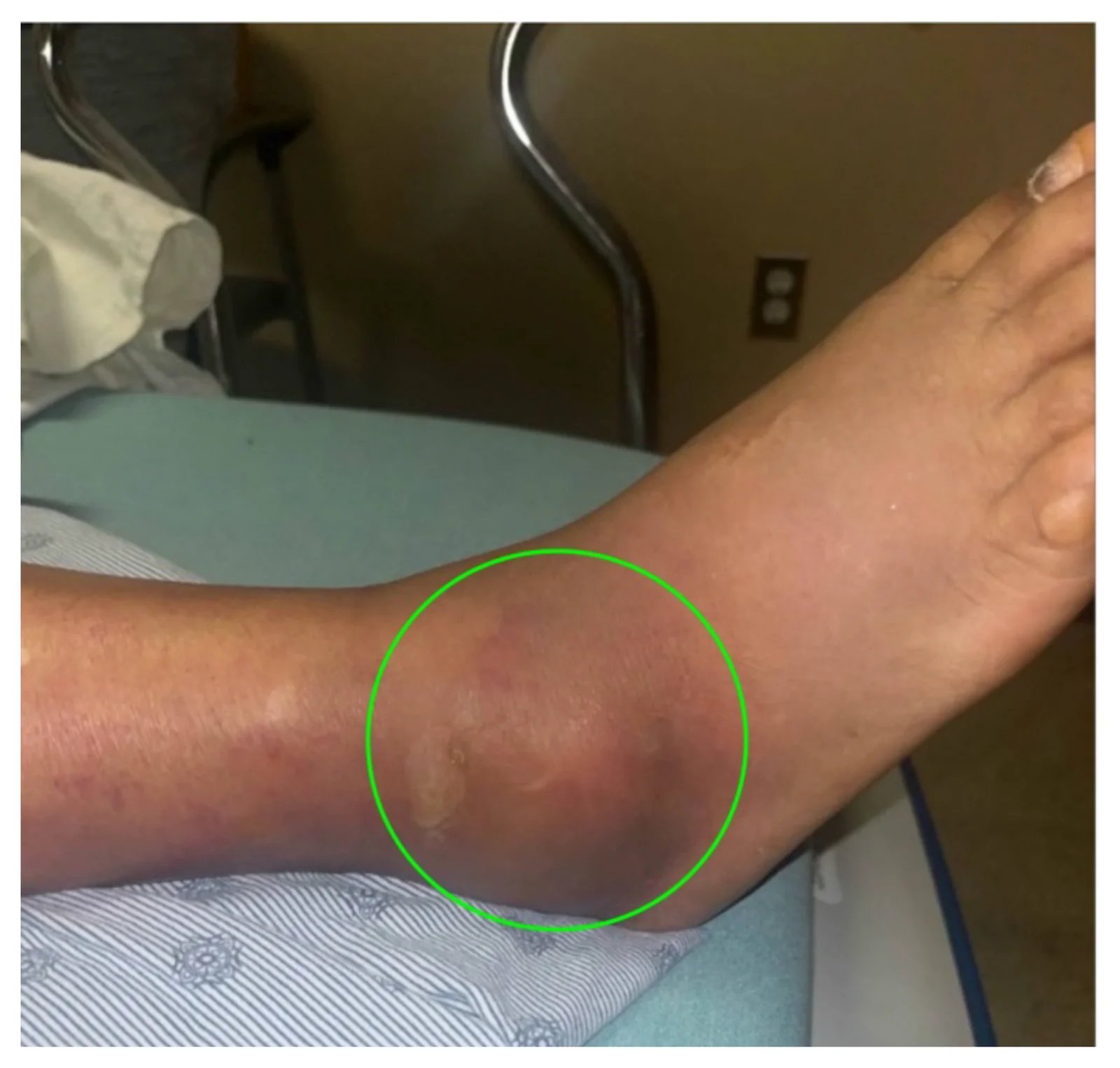


**Figure:**
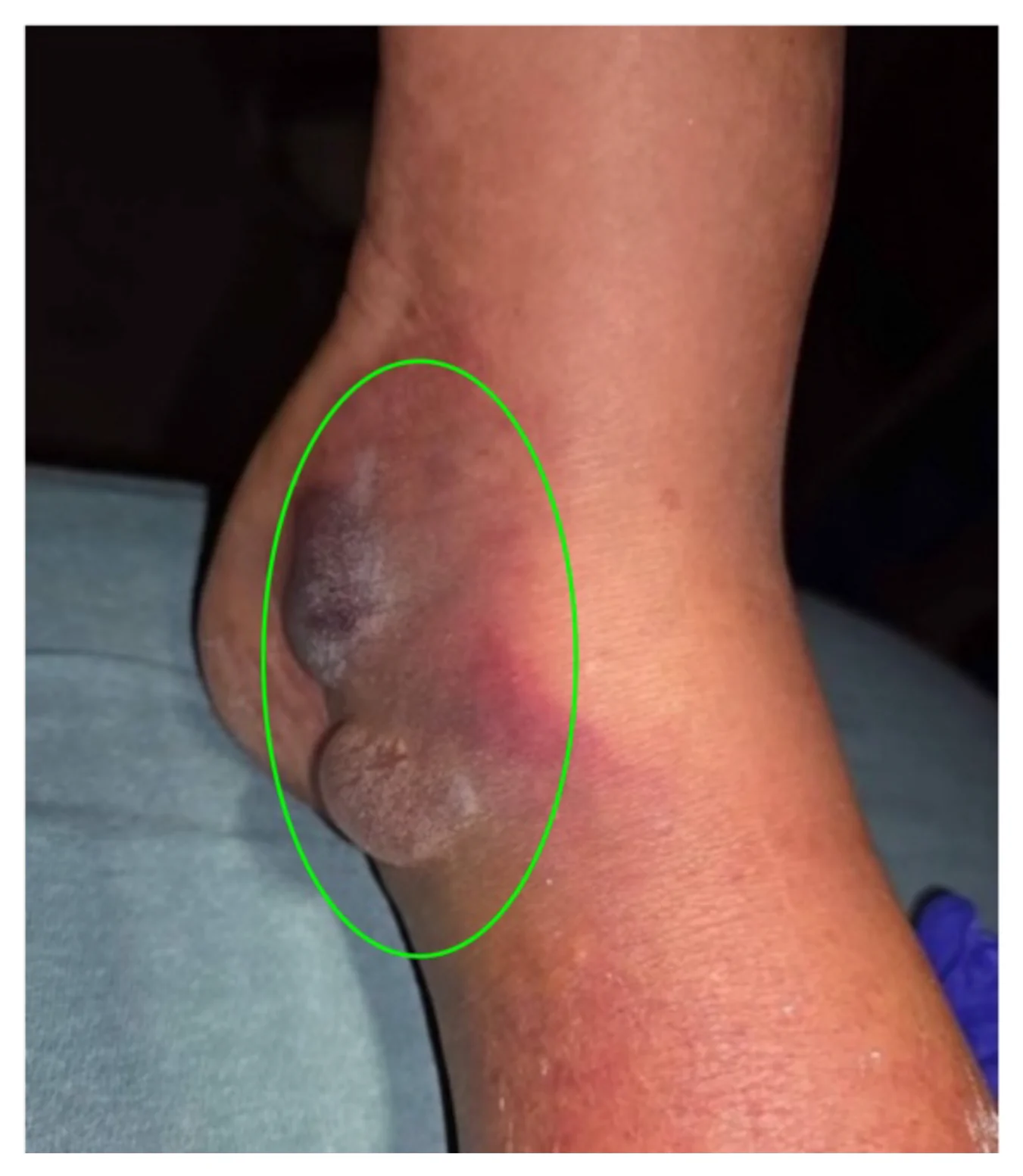


**Figure:**
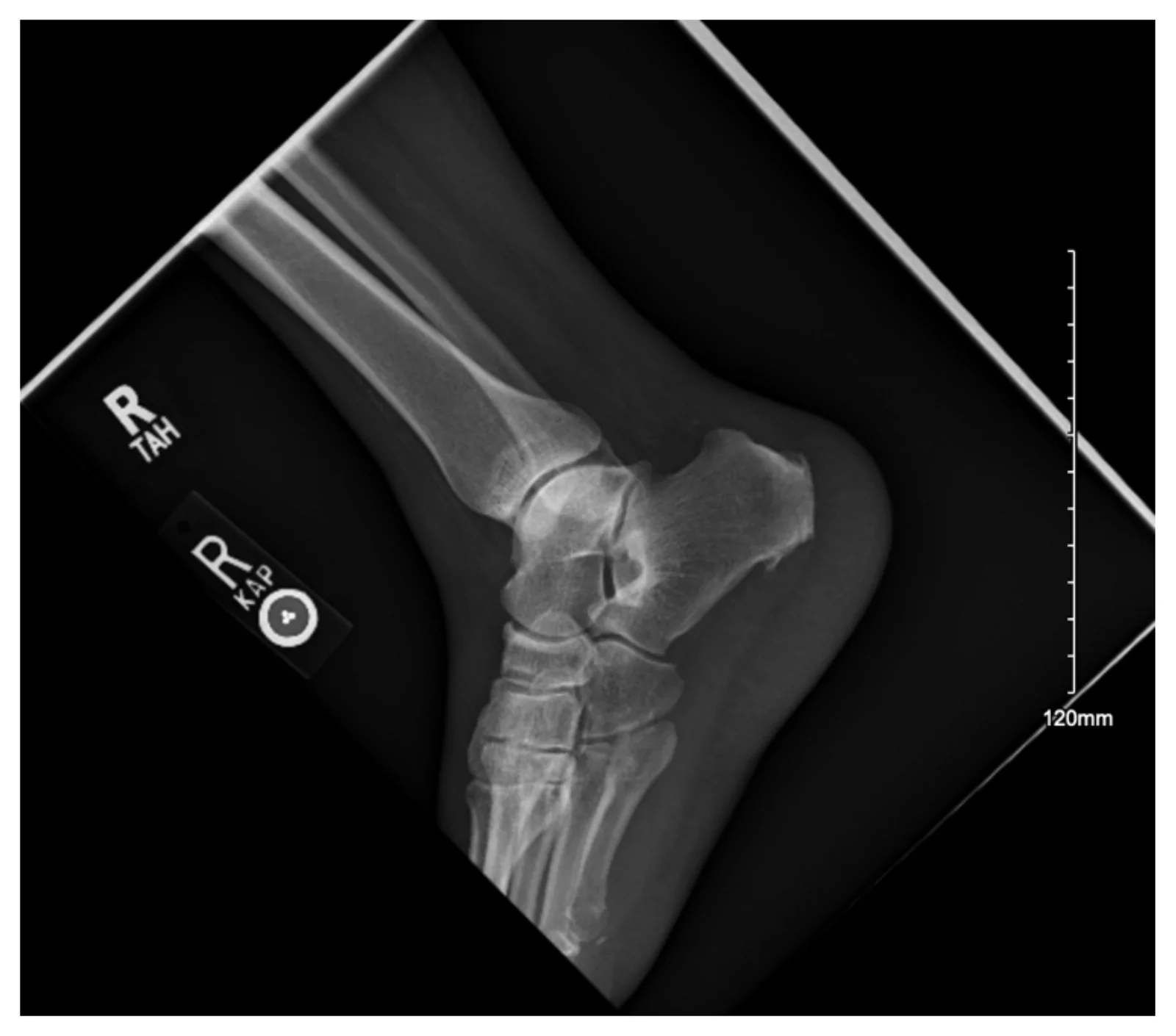


**Figure:**
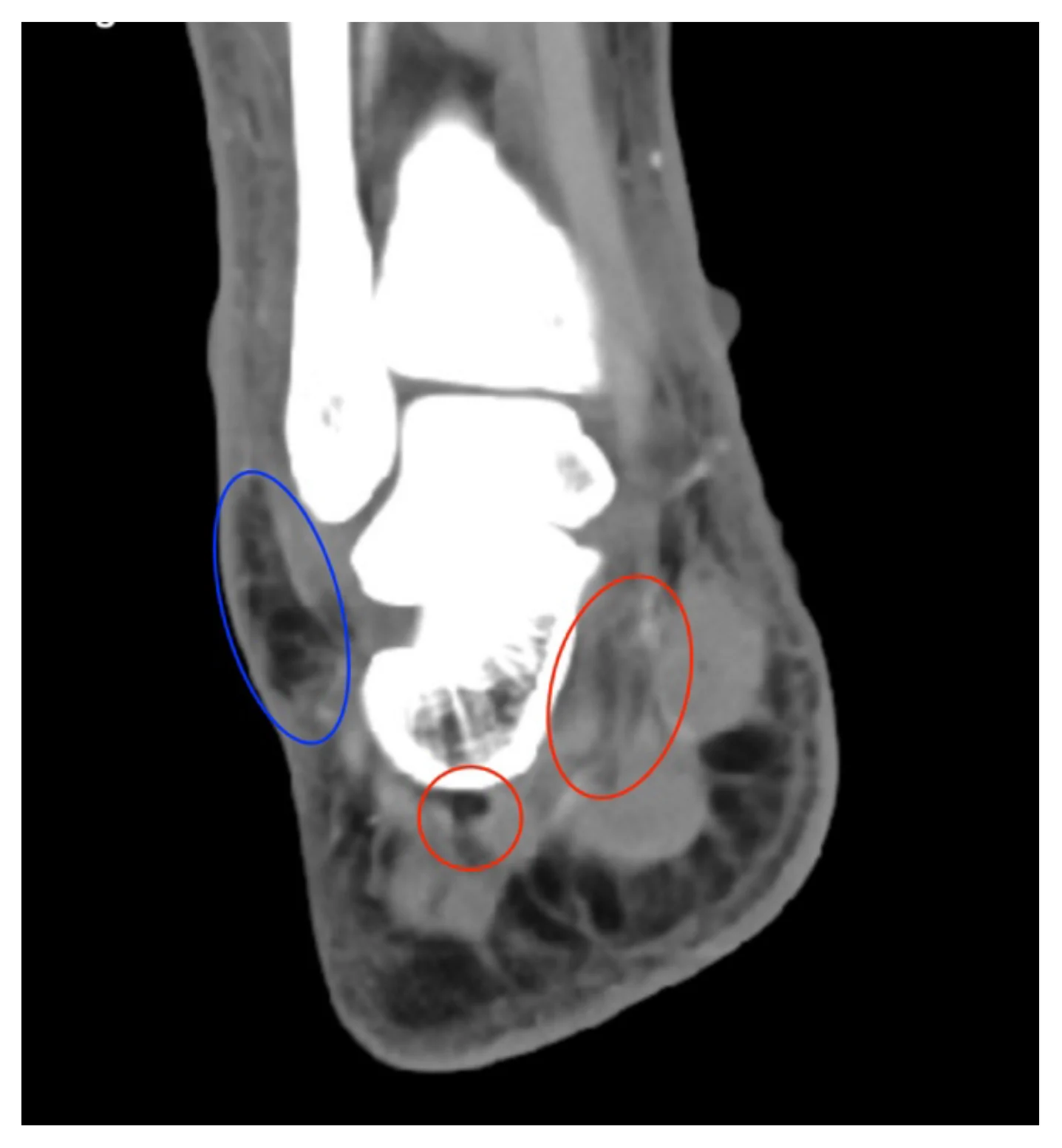


**Figure:**
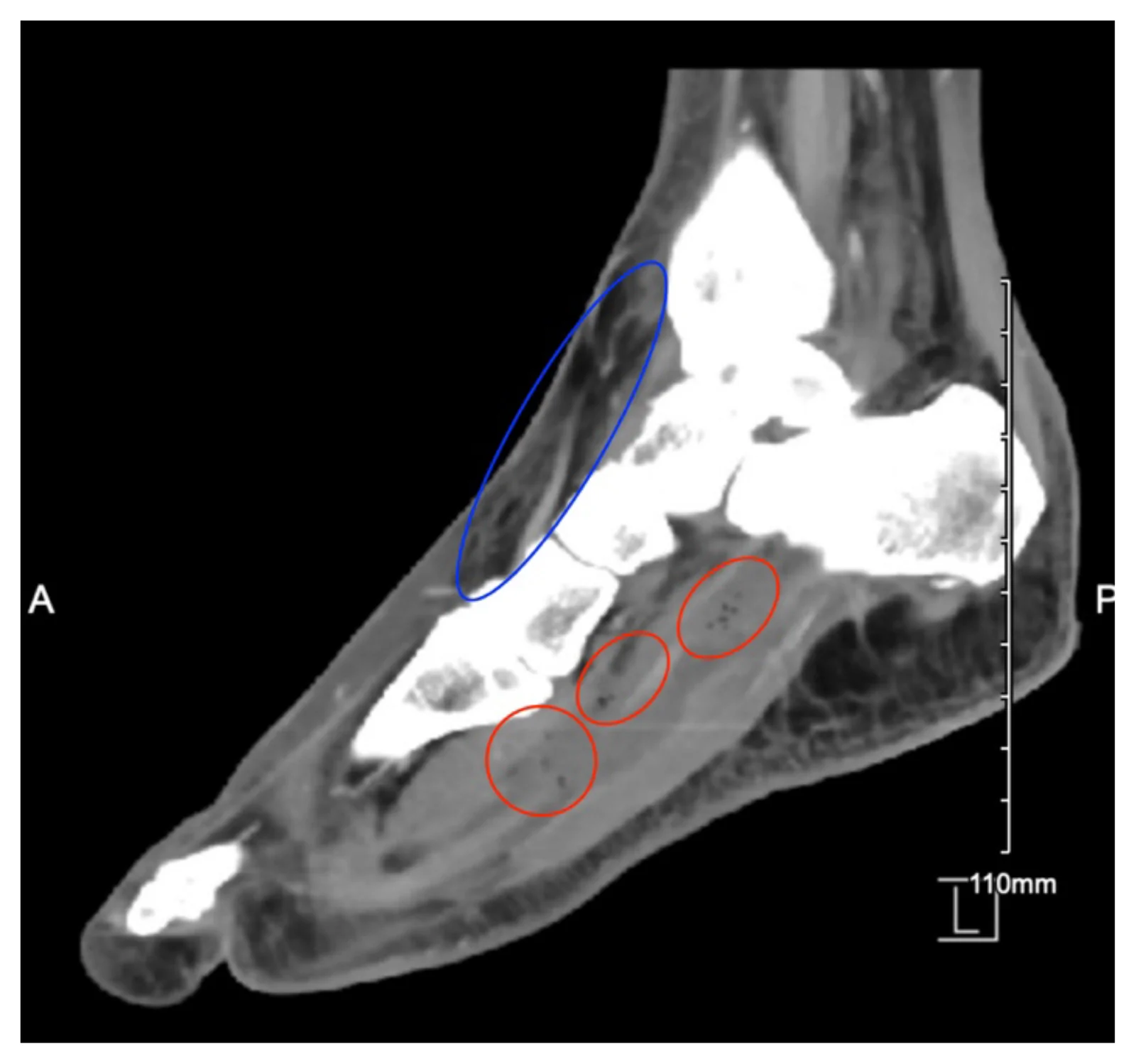


## Brief Introduction

Infectious flexor tenosynovitis (IFT) is an infection of the tendon sheath caused by microbial invasion. While commonly associated with penetrating trauma or hematogenous spread, IFT is a severe condition that can lead to devastating complications if not promptly recognized and treated.^1^–^4^ The most common organisms found to cause IFT include *Staphylococcus aureus* (40% to 75%) and *MRSA* (29%).^5^ Incidence of the stenosing form of tenosynovitis in the general population is 1.7–2.6%; however, this value is much higher in patients with diabetes mellitus, increasing to 10–20% incidence.^6^ The flexor hallucis longus (FHL) and flexor digitorum longus (FDL) tendons play a critical role in foot movement and weight-bearing, making infections in these structures particularly concerning.

In this case, we describe a patient with infectious tenosynovitis involving both the FHL and FDL tendons, complicated by extensive soft tissue necrosis. Given the patient's history of chronic medical conditions, including diabetes and cirrhosis, this case highlights the increased susceptibility to severe infections and the challenges in managing complex soft tissue infections in immunocompromised individuals.

## Presenting concerns and clinical findings

A 57-year-old female, with a past medical history of cirrhosis secondary to hepatitis C virus infection, type 2 diabetes mellitus, and hypertension, presented to the emergency department (ED) with a two-week history of right ankle pain, which worsened acutely over the past day. Approximately one month ago, the patient sustained an injury to her right ankle when she missed a step and twisted it. Following this incident, she had visited the emergency department on two occasions, during which she was discharged with pain medications and an orthopedic walking boot.

During her third visit, she reported worsening throbbing ankle pain with swelling, erythema, bruising, subjective fevers, chills, and nausea. She was unable to bear weight on the affected extremity. She denied experiencing chest pain, shortness of breath, or vomiting. Conservative measures, including ice, elevation, and analgesics, provided minimal relief.

Examination revealed blistering and erythema over the medial and lateral right ankle and foot (Image 1–3, green highlighted). The patient demonstrated significant discomfort even with light touch applied to her lower extremity. Vital signs reflected tachycardia with a heart rate of 103 beats per minute (BPM); however, she remained hemodynamically stable overall. Laboratory results indicated a glucose level of 473 mg/dL, a creatinine level of 1.03 mg/dL, a blood urea nitrogen (BUN) level of 53 mg/dL, leukocytosis of 16.83 x 10^9^/L, a C-reactive protein (CRP) level of 18.8 mg/L, and a lactate level of 3.4 mmol/L. Subsequently, X-rays of the foot, ankle, and tibia-fibula, a contrast-enhanced computed tomography (CT) scan of the right lower extremity with intravenous (IV) contrast, and a deep vein thrombosis (DVT) ultrasound study were ordered to further evaluate the patient. Written consent was obtained by the patient to use this clinical information for medical teaching and publishing.

## Significant findings

X-rays of the foot, ankle, and tibia-fibula were obtained which showed no acute fractures or dislocations. A CT of the right lower extremity with IV contrast demonstrated fluid along the flexor hallucis longus and flexor digitorum longus tendons with scattered foci of air, and the hypodensities on the plantar aspect of the foot (red highlight), were concerning for infectious tenosynovitis. The CT scan also showed diffuse soft tissue edema, the dark attenuation on imaging (blue highlighted), predominantly along the dorsal midfoot.

## Patient course

After the CT scan results showed a fluid collection along the flexor hallucis longus and flexor digitorum longus tendons, podiatry was consulted for further management. Broad spectrum antibiotics (vancomycin, clindamycin, and piperacillin-tazobactam). This antibiotic regimen was chosen to target MRSA, Group A streptococci, and gram-negative bacteria. The patient had a negative ultrasound scan for DVT. Podiatry assessed the patient and planned an incision and drainage with exploration. They had high concerns for deep soft tissue necrosis and infection that could span the entire forefoot and ankle. The patient was taken to the operating room (OR) for exploration and found to have extensive liquefactive necrosis of the skin, subcutaneous tissue, purulence in ankle/subtalar joint along the peroneal tendons and along flexor tendons from level of midfoot to proximal calf. The foot was not salvageable, and general surgery was consulted to complete a guillotine below-the knee amputation (BKA). Blood cultures obtained grew *group B streptococcus*. Cultures from the wound grew gram positive cocci. The patient’s antibiotics were narrowed to Rocephin for 14 days. On postoperative day (POD) 6, the patient was taken back to the OR for formalization of right BKA. The patient’s postoperative course was complicated by neuropathic pain, hepatic encephalopathy, and hematochezia which dropped her hemoglobin to 6.9 from 9 and required a blood transfusion. However, she was ultimately discharged with follow up with podiatry, general surgery, and hepatology.

## Discussion

IFT is an infection of the synovial tendon sheath that most commonly affects the flexor tendons of the hand; lower extremity involvement is rare^7^. The enclosed tendon sheath facilitates pathogen proliferation and limits immune clearance.^8^

Kanavel’s signs are the classic clinical indicators of IFT and demonstrates high sensitivity (91%–97%) but low specificity (51%–69%).^10^–^11^

Treatment decisions for IFT, including the need for surgical intervention, depend on the stage of infection. IFT progresses from exudative inflammation (Stage 1) to purulent infection (Stage 2) and septic necrosis with tendon sheath destruction (Stage 3). Surgical debridement is typically required for Stages 2 and 3.^12^–^13^

Prior studies have shown that delayed surgical debridement increases the risk of extensive tissue loss and amputation, particularly among diabetic patients, findings that are consistent with this case.^4^,^16^ Additionally, the role of broad-spectrum intravenous antibiotics, followed by culture-directed therapy, is well supported in the literature as an essential component of treatment. 13–15 It's important to note that other conditions can mimic flexor tendon issues in the foot. For example, some literature discusses metatarsalgia, which can cause pain in the ball of the foot or tendinopathy of the foot related to antibiotics. 16,17 While not directly related to tenosynovitis, it's a crucial differential diagnosis to consider since these other pathologies do not have significant morbidity and mortality.^16^

This case report highlights the diagnostic and management challenges associated with IFT of the lower extremity, particularly in an immunocompromised patient with multiple comorbidities. The strengths of this case include the multidisciplinary approach to management, the use of advanced imaging modalities for early diagnosis, and the detailed documentation of disease progression leading to limb amputation.

Lower-extremity IFT is rare and may be difficult to recognize early. In this case, delayed diagnosis despite multiple healthcare encounters allowed progression to extensive soft tissue necrosis. Although CT findings were highly suggestive of infection, microbiologic confirmation remains important when imaging findings are equivocal. Ultimately, this case underscores the aggressive nature of infectious tenosynovitis in immunocompromised patients and highlights the importance of early diagnosis and prompt surgical intervention to improve clinical outcomes.^10^–^12^

## Supplementary Information
























